# Effect of dose constraint on the thyroid gland during locoregional intensity‐modulated radiotherapy in breast cancer patients

**DOI:** 10.1002/acm2.12668

**Published:** 2019-06-24

**Authors:** Emel Haciislamoglu, Emine Canyilmaz, Sonay Gedik, Ozlem Aynaci, Lasif Serdar, Adnan Yoney

**Affiliations:** ^1^ Department of Radiation Oncology, Faculty of Medicine Karadeniz Technical University Trabzon Turkey; ^2^ Department of Radiation Oncology Kanuni Research and Education Hospital Trabzon Turkey

**Keywords:** breast cancer, dose constraint, supraclavicular radiotherapy, thyroid gland dose

## Abstract

The aim of the present study was to compare radiation dose received by thyroid gland using different radiotherapy (RT) techniques with or without thyroid dose constraint (DC) for breast cancer patients. Computerized tomography (CT) image sets for 10 patients with breast cancer were selected. All patients were treated originally with opposite tangential field‐in field (FinF) for the chest wall and anteroposterior fields for the ipsilateral supraclavicular field. The thyroid gland was not contoured on the CT images at the time of the original scheduled treatment. Four new treatment plans were created for each patient, including intensity‐modulated radiotherapy (IMRT) and helical tomotherapy (HT) plans with thyroid DC exclusion and inclusion (IMRT_DC(−)_, IMRT_DC(+)_, HT_DC(−)_, and HT_DC(+)_, respectively). Thyroid DCs were used to create acceptable dose limits to avoid hypothyroidism as follows: percentage of thyroid volume exceeding 30 Gy less than 50% (*V*_30_ < 50%) and mean dose of thyroid (TD_mean_) ≤ 21 Gy. Dose‐volume histograms (DVHs) for TD_mean_ and percentages of thyroid volume exceeding 10, 20, 30, 40, and 50 Gy (*V*_10_, *V*_20_, *V*_30_, *V*_40_, and *V*_50_, respectively) were also analyzed. The D_mean_ of the FinF, IMRT_DC(−)_, HT_DC(−)_, IMRT_DC(+)_ and HT_DC(+)_ plans were 30.56 ± 5.38 Gy, 25.56 ± 6.66 Gy, 27.48 ± 4.16 Gy, 18.57 ± 2.14 Gy, and 17.34 ± 2.70 Gy, respectively. Median V_30_ values were 55%, 33%, 36%, 18%, and 17%, for FinF, IMRT_DC(−)_, HT_DC(−)_, IMRT_DC(+)_, and HT_DC(+)_, respectively. Differences between treatment plans with or without DC with respect to *D*_mean_ and *V*_30_ values were statistically significant (*P < *0.05). When thyroid DC during breast cancer RT was applied to IMRT and HT, the TD_mean_ and *V*_30_ values significantly decreased. Therefore, recognition of the thyroid as an organ at risk (OAR) and the use of DCs during IMRT and HT planning to minimize radiation dose and thyroid volume exposure are recommended.

## 1 INTRODUCTION

Breast cancer is the second most common cancer nowadays, after lung cancer.^1^ Surgery is one of the most clinically beneficial procedures for treatment of breast cancer. However, it is possible that after surgery the remaining deposits of neoplastic disease locally or at distant sites are present.^2^ Therefore, radiotherapy (RT) plays an important role in removal of the resident deposit of breast cancer.^3^, ^4^ Unfortunately, the side effects of RT are inevitable, particularly on the sensitive organs such as thyroid gland.^5^, ^6^

Thyroid gland is very sensitive, important and the largest pure endocrine gland in our body and more importantly its hormones play a very significant role in metabolism, development, growth, overall energy expenditure, and a large number of body organs functions.^7^, ^8^ Primary hypothyroidism is a well known side effect of curative RT in patients with head and neck cancer and Hodgkin lymphoma,^9^, ^10^, ^11^, ^12^ whose RT portals usually encompass the entire thyroid.^13^, ^14^, ^15^, ^16^ However, limited data are available regarding hypothyroidism in patients with breast cancer treated with locoregional RT wherein the treatment field includes only part of the thyroid.^17^, ^18^, ^19^, ^20^, ^21^

Many studies have shown that radiation can cause thyroid gland disorders,^7^, ^8^, ^11^, ^22^ although the tolerance dose (TD) of the thyroid gland has not been definitively established.^23^ The minimum thyroid TD_5/5_ (incidence of clinical hypothyroidism in 5% of patients at 5 yr after treatment) is considered to be 20 Gy when all or part of the gland is irradiated with conventional fractionation.^12^, ^24^ Although some studies have reported the occurrence of RT‐induced hypothyroidism at high radiation doses (e.g., ≥30 Gy),^21^, ^25^ Dorri et al.^3^ observed no significant differences in thyroid hormone levels before and after RT in breast cancer patients, further highlighting the contradictory findings regarding RT’s effects on thyroid function.

Our knowledge of radiation‐induced hypothyroidism in patients with breast cancer is limited because the thyroid gland is not routinely considered as an organ at risk (OAR) during the irradiation of breast cancer. There is a growing body of literature examining the relationship between thyroid dose and hypothyroidism development in breast cancer RT.^26^, ^27^ However, few studies have compared the effects of different RT techniques on the thyroid dose.

The purpose of this study was to dosimetrically compare locoregional breast treatment plans using tangential field‐in‐field (FinF), intensity‐modulated radiotherapy (IMRT) and helical tomotherapy (HT) techniques in terms of thyroid dose that could potentially predict RT‐induced hypothyroidism risk and to determine whether the use of thyroid dose constraint (DC) is beneficial.

## 2 MATERIALS AND METHODS

### 2.A Computerized tomography (CT) imaging

Computerized tomography (CT) image sets for 10 patients with breast cancer were selected from our treatment database. All patients underwent our department's routine procedures for patients with breast cancer. During the CT scan, each patient was in a supine position on a breast board, adjusted to achieve a flat chest wall with the head turned away from the side of treatment and the ipsilateral arm placed above the head.

### 2.B Target delineation

The chest wall and ipsilateral supraclavicular field (SCF) were delineated for each patient by an experienced radiation oncologist as a clinical target volume (CTV), along with the contralateral breast, spinal cord, heart, and both lungs. The SCF included the supraclavicular (SC) and level‐1,2,3 axillary nodes. Consensus guidelines of the Radiation Therapy Oncology Group were used to delineate the CTV of the chest wall and SCF. The planning target volume (PTV) was created by adding 5 mm to the CTV. The thyroid gland was not contoured on the CT images at the time of the original scheduled treatment. For this study, the same physician manually contoured the thyroid gland on the CT‐simulated images of all patients.

### 2.C Design of the treatment plans

For each of the ten patients, five different plans were created: field‐in‐field (FinF), intensity‐modulated radiotherapy (IMRT) thyroid DC exclusion IMRT_DC(−)_, IMRT thyroid DC inclusion IMRT_DC(+)_, helical tomotherapy (HT) thyroid DC exclusion HT_DC(−)_, and HT thyroid DC inclusion HT_DC(+)_. All patients were treated originally with opposite tangential field‐in‐field (FinF) for the chest wall and anteroposterior fields for the ipsilateral supraclavicular field (SCF). The prescribed dose was 50 Gy in 25 fractions, 5 days per week.

For the FinF technique, the beam arrangement consisted of two parallel opposing tangential beams to ensure the best possible coverage of the chest wall tissue and anteroposterior fields (with 15°–250° gantry angles) for the ipsilateral SCF. A single isocenter was chosen at the level of the match line between the ipsilateral SCF and chest wall below the medial end of the clavicle. Photon energy of 6 MV was used for both the tangential fields and anterior fields of the SCF; 18 MV was used for posterior fields. Shielding blocks were used primarily for spinal cord; no attempt was made to shield thyroid gland itself to prevent any under dosage in SCF.

The IMRT plans consisted of nine coplanar beams. The lateral and medial gantry angles were the same as those used in the FinF approach, while the other seven fields were placed between these fields at equal intervals. The field width, pitch, and modulation factor parameters were assigned as 2.5 cm, 0.287, and 2.0, respectively, for the HT plans. Two virtual structures (constraint‐lung and constraint‐heart) for DCs were contoured for each patient to decrease radiation doses to the lungs and heart. Partial blocking was applied to the contralateral breast.

The FinF and IMRT plans were generated using the Eclipse™ treatment planning system (Varian Medical Systems, Palo Alto, CA) and the HT plans were performed using a tomotherapy Hi‐ART planning system. The dose‐volume constraints used for the IMRT and HT plans are presented in Table 1. While DCs were applied for the heart, ipsilateral lung, contralateral lung, and contralateral breast in the IMRT_DC(−)_ and HT_DC(−)_ plans, thyroid DCs were included in the IMRT_DC(+)_ and HT_DC(+)_ plans in addition to the above constraints.

**Table 1:** Target doses and dose constraints (DCs) of the organs at risk (OARs).

Target or OAR	Goal or constraint dose
Planning target volume	45 or 47.5 Gy
Heart	*V*_20_ < 10%
İpsilateral lung	*V*_20_ < 35%
Contralateral lung	*V*_5_ < 20%
Contralateral breast	*D*_max_ < 10 Gy
*Thyroid “IMRT*_*DC*(+)_ *and HT*_*DC*(+)_ *plans with thyroid dose constraint (DC)*”	*D*_mean_ ≤ 21 Gy; *V*_30_ < 50%

### 2.D Dose‐volume histogram data and statistical analysis

The generated treatment plans were compared objectively using dose‐volume histograms (DVHs) for PTVs and different OARs of interest. In the PTV, mean dose (D_mean_), conformation number (CN), and homogeneity index (HI) were compared between all five plans. CN is calculated from the following formula:

$$\text{CN}=\left(\text{TVRI}/\text{TV}\right)\left(\text{TVRI}/\text{VRI}\right)$$

where TVRI is the target volume covered by the reference isodose (95% of the prescribed dose), TV is the target volume, and VRI is the volume of the reference isodose. The CN ranges from 0 to 1, where 1 is the ideal value.

Another index for evaluating the plan is the HI, which takes into the homogeneity of the dose distribution within the target. HI is calculated from the following formula:

$$\text{HI}=\left(D_{2}-D_{98}/D_{50}\right)\times 100\%$$

where *D*_98_ for the PTV is the corresponding dose for 98% of the target volume measured on DVH, and *D*_2_ is the corresponding dose for 2% of the volume on the DVH. HI formula shows that lower HI values indicate a more homogeneous target dose.

Based on each patient's dose‐volume histograms (DVHs), TD_mean_ values and the percentage of thyroid gland volume that received 10 Gy (*V*_10_), 20 Gy (*V*_20_), 30 Gy (*V*_30_), 40 Gy (*V*_40_), and 50 Gy (*V*_50_) were analyzed. Additionally, when using DC to the thyroid gland in the IMRT_DC(+)_ and HT_DC(+)_ plans, *V*_45_ of the SC node, which is very close to the thyroid, was evaluated.

Statistical Package for the Social Sciences (SPSS) version 18 for Windows software was used for statistical analysis. Post hoc ANOVA was used to compare parametric data; nonparametric data were analyzed with Kruskal‐Wallis tests. For paired group comparisons of quantitative data, the Bonferroni modified test was applied for parametric data, while the Mann‐Whitney *U* test was used for nonparametric data. Differences were considered significant at *P* ≤ 0.05.

## 3 RESULT

The doses of planning target volume and OAR according to five different plans are summarized in Table 2.

**Table 2:** Comparision of target coverage metrics for the planning target volume (PTV) and organs at risk (OAR) dose‐volume metrics as a function of plan modality ($\overset{¯}{x}$ ± SD).

Metric	FinF	IMRT_DC(−)_	IMRT_DC(+)_	HT_DC(−)_	HT_DC(+)_	*P*‐Value
PTV						
*D*_mean_ (Gy)	51.56 ± 1.00	51.24 ± 0.37	51.31 ± 0.37	50.83 ± 0.21	50.88 ± 0.21	0.005
CN	0.61 ± 0.09	0.76 ± 0.04	0.76 ± 0.04	0.80 ± 0.03	0.80 ± 0.03	<0.001
HI	0.12 ± 0.05	0.08 ± 0.01	0.08 ± 0.01	0.06 ± 0.01	0.06 ± 0.01	<0.001
Heart						
*D*_mean_ (Gy)	4.30 ± 2.22	8.42 ± 2.51	8.49 ± 2.52	4.17 ± 0.78	4.25 ± 0.77	<0.001
*V*_20_ (%)	5.2 ± 4.32	2.1 ± 1.65	2.15 ± 1.64	0.1 ± 0.14	0.12 ± 0.14	<0.001
Ipsilateral lung						
*D*_mean_ (Gy)	7.35 ± 2.42	12.24 ± 2.21	12.29 ± 2.20	5.18 ± 1.35	5.24 ± 1.34	<0.001
*V*_20_ (%)	12.65 ± 4.80	15.10 ± 5.37	15.15 ± 5.36	7.20 ± 2.30	7.27 ± 2.31	<0.001
Contralateral lung						
*D*_mean_ (Gy)	0.40 ± 0.20	4.21 ± 1.10	4.23 ± 1.11	2.52 ± 0.86	2.55 ± 0.87	<0.001
*V*_5_ (%)	0.0 ± 0.0	21.75 ± 14.43	21.81 ± 14.40	19.16 ± 11.60	19.21 ± 11.53	<0.001
Contralateral breast						
*D*_max_ (Gy)	2.82 ± 0.70	9.10 ± 3.32	9.15 ± 3.31	9.88 ± 2.06	9.86 ± 2.05	<0.001

PTV, Planning Target Volume; *D*_max_, max dose; *D*_mean_, mean dose; *V*_x_, volume (%) receiving × dose (Gy) or higher; $\overset{¯}{x}$, mean dose; sd, standart deviation; CN, conformation number; HI, homogeneity index.

The mean thyroid gland volume of 10 patients was 11.9 cm^3^ (6.3–19.8 cm^3^). Detailed dosimetric results for the thyroid glands and SC nodes for the five different plans are presented in Table 3.

**Table 3:** Comparison of thyroid gland and supraclavicular (SC) node dosimetric parameters as a function of treatment plans.

Metric	FinF	IMRT_DC(−)_	HT_DC(−)_	IMRT_DC(+)_	HT_DC(+)_	*P*‐value
*D*_mean_ (Gy)	30.56 ± 5.38	25.56 ± 6.66	27.48 ± 4.16	18.57 ± 2.14	17.34 ± 2.7	**<0.001**
*V*_10_ (%)	67 ± 10.51	92 ± 13.82	96 ± 5.93	76 ± 11.92	70 ± 10.55	**<0.001**
*V*_20_ (%)	60 ± 10.03	56 ± 19.58	66 ± 16.31	31 ± 7.03	28 ± 11.22	**<0.001**
*V*_30_ (%)	55 ± 10.81	33 ± 16.81	36 ± 14.27	18 ± 7.09	17 ± 9.74	**<0.001**
*V*_40_ (%)	51 ± 11.76	22 ± 16.41	21 ± 14.2	8 ± 6.48	7 ± 8.92	**<0.001**
*V*_50_ (%)	30 ± 15.59	7 ± 7.64	4 ± 6.04	1 ± 3.08	2 ± 4.64	**<0.001**
SC Node *V*_45_ (%)	100	99.2 ± 0.53	100	98.6 ± 0.83	97.9 ± 0.66	**<0.001**

*D*_mean_, mean dose; Gy, Gray; *V*_x_, volume (%) receiving × dose (Gy) or higher. Values in bold font are statistically significant. Mean ± SD values are presented.

Significant differences were observed between plans with respect to TD_mean_ (*P* < 0.001). The TD_mean_ ± standard deviation values for the FinF, IMRT_DC(−)_, HT_DC(−)_, IMRT_DC(+)_, and HT_DC(+)_ plans were 30.56 ± 5.38 Gy, 25.56 ± 6.66 Gy, 27.48 ± 4.16 Gy, 18.57 ± 2.14 Gy, and 17.34 ± 2.70 Gy, respectively.

**Figure 1:**
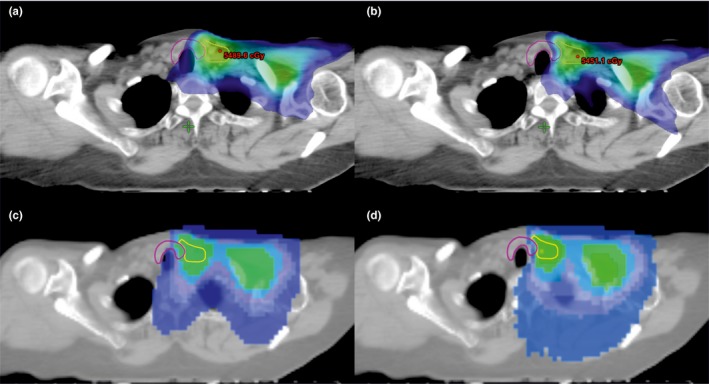
The isodose distribution for the four plans in axial plane for a representative patient. Color‐wash threshold was set to 21 Gy. (a) IMRT_DC(−)_; (b) IMRT_DC(+);_ (c) HT_DC(−)_; and (d) HT_DC(+)_. IMRT, intensity modulated radiotherapy; HT, helical tomotherapy.

The TD_mean_ for the FinF, IMRT_DC(2212)_, and HT_DC(‐)_ plans was >21 Gy, while the TD_mean_ for the IMRT_DC(+)_ and HT_DC(+)_ plans was <21 Gy. Figure 1 shows the isodose distribution for the IMRT_DC(−)_, IMRT_DC(+)_, HT_DC(‐)_ and HT_DC(+)_ plans in axial plane for a representative patient. The color‐wash threshold was set to 21 Gy. IMRT_DC(−)_ and HT_DC(−)_ plans reduced TD_mean_ values from those used in FinF, while IMRT_DC(+)_ and HT_DC(+)_ further reduced the TD_mean_.

**Figure 2:**
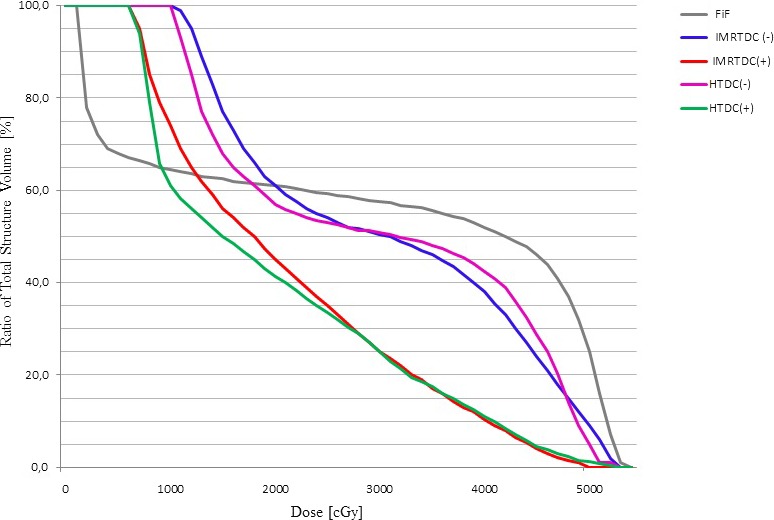
Dose‐volume histograms (DVH) comparison of the thyroid gland using FinF, IMRT_DC(−)_, HT_DC(−)_, IMRT_DC(+)_, and HT_DC(+)_ in a representative patient. IMRT, intensity modulated radiotherapy; HT, helical tomotherapy.

There was no statistically significant difference between the three plans [FinF, IMRT_DC(−)_, and HT_DC(−)_] with respect to TD_mean_ (*P*> 0.05; Table 4). However, the TD_mean_ values for the IMRT_DC(+)_ and HT_DC(+)_ plans were significantly lower than those of the other three plans. Differences between IMRT_DC(+)_ and HT_DC(+)_ TD_mean_ values were not statistically significant (*P* = 0.958); in contrast, TD_mean_ difference significantly between the DC(_−_) and DC(+) plans (*P* < 0.001). An illustrative DVH comparison for thyroid gland for a representative patient is shown in Fig. 2.

**Table 4:** Estimated *P*‐values for the compared treatment plans.

Metric	Thyroid						SC Node *V*_45_(%)
	*D*_mean_	*V*_10_(%)	*V*_20_(%)	*V*_30_(%)	*V*_40_(%)	*V*_50_(%)	
FinF vs IMRT_DC(_ *_2212_*_)_	0.576	**<0.001**	0.950	**0.002**	**0.004**	**0.010**	**0.012**
FinF vs IMRT_DC(+)_	**<0.001**	0.304	**<0.001**	**<0.001**	**<0.001**	**0.002**	**0.004**
FinF vs HT_DC(−)_	0.846	**<0.001**	0.984	**0.007**	**<0.001**	**0.004**	**<0.001**
FinF vs HT_DC(+)_	**<0.001**	0.946	**<0.001**	**<0.001**	**<0.001**	**0.002**	**<0.001**
IMRT_DC(−)_ vs IMRT_DC(+)_	***<0.001***	**0.015**	**0.041**	**0.047**	0.232	0.439	0.408
IMRT_DC(−)_ vs HT_DC(−)_	0.997	0.915	0.634	0.989	1.000	0.982	**0.012**
IMRT_DC(−)_ vs HT_DC(+)_	**0.035**	**<0.001**	**0.049**	**0.043**	0.185	0.646	**0.002**
IMRT_DC(+)_ vs HT_DC(−)_	**<0.001**	**<0.001**	**<0.001**	**0.014**	0.235	0.960	**0.004**
IMRT_DC(+)_ vs HT_DC(+)_	0.958	0.743	0.998	1.000	1.000	1.000	0.577
HT_DC(−)_ vs HT_DC(+)_	**<0.001**	**<0.001**	**<0.001**	**0.012**	0.189	0.997	**<0.001**

Values in bold font are statistically significant.

The low dose‐volume (V_10_) in the thyroid gland was larger for the IMRT and HT plans compared with the FinF plan. It was found that the volume percentage of the thyroid absorbing ≥30 Gy was above 50% in seven of 10 in patients in FinF and two of ten both IMRT_DC(−)_ and HT_DC(−)._

However, the mean dose for *V*_30_ was <50% for the IMRT_DC(−)_ and HT_DC(−)_ plans (33% and 36%, respectively). When DC was applied for both IMRT and HT, *V*_30_ ≥ 50% was not observed for any patient. The differences between the DC(_−_) and DC(+) plans were statistically significant for *V*_10_, *V*_20_, and *V*_30_. The *V*_30_ values for the IMRT_DC_(+) and HT_DC(+)_ plans were significantly lower than the other three plans. There were no statistically significant differences between *V*_10_, *V*_20_, *V*_30_, *V*_40_, and *V*_50_ values for the IMRT_DC(+)_ and HT_DC(+)_ plans.

We found no statistically significant difference between the IMRT_DC(−)_ and IMRT_DC(+)_ plans with respect to the SC node V_45_ value. Although the SC node V_45_ values were significantly different in the HT_DC(−)_ and HT_DC(+)_ plans, 97.9% of the SC node volume was covered by 90% of the prescribed dose (45 Gy) for HT_DC(+)_ plans (Table 3).

## 4 DISCUSSION

The thyroid gland is very sensitive to radiation and a large number of studies showed that radiation can cause disorders such as hypothyroidism, Graves' disease, and thyroid cancer.^7^, ^8^, ^11^ Although the dose of radiation is a significant factor for predicting thyroid dysfunction,^21^, ^28^, ^29^, ^30^, ^31^ few investigators have performed clinical thyroid‐associated DVH analysis after RT.^12^, ^32^, ^33^ Most of these investigations were studied in patients with head and neck cancer patients treated with RT doses higher than those used in RT for breast cancer.^12^, ^26^, ^31^

Hypothyroidism is one of the late toxicities of curative RT to the neck region, and the incidences of hypothyroidism that have been reported range from 20% to 52%.^9^, ^10^, ^11^, ^12^, ^29^ Unfortunately, our knowledge of radiation‐induced hypothyroidism in breast cancer patients is limited because the thyroid gland is not routinely considered as an OAR during breast cancer RT. As a result, radiation‐induced hypothyroidism in these patients has been investigated in only a few studies, which reported varying incidence rates (6%–21%) in patients with breast cancer.^17^, ^19^, ^20^, ^21^, ^34^

The correlation between radiation dose and hypothyroidism was demonstrated by Kuten et al.^9^ and Yoden et al.,^28^ who used DVHs to evaluate the relationship between the volume of the thyroid receiving radiation and thyroid function. Their results indicated that the thyroid volume receiving doses *V*_10_ to *V*_30_ significantly impacted the peak level of thyroid‐stimulating hormone. Similarly, Cella et al.^32^ and Akgun et al.^29^ reported that V_30_ was a statistically significant predictor for the development of hypothyroidism. According to Kanyılmaz et al.,^27^ D_mean_ was the only factor that accurately predicted hypothyroidism, with 21 Gy as the threshold value. Additionally, Tunio et all.^26^ showed that the risk of hypothyroidism in breast cancer patients after SC‐RT depends on the thyroid gland volume and *V*_30_ > 50%.

In contrast, Diaz et al.^35^ reported that the *D*_mean_ and *V*_10_ to *V*_70_ were not associated with hypothyroidism. Alterio et al.^12^ also showed that *D*_mean_, *V*_10_, *V*_30_, and *V*_50_ were not associated with hypothyroidism, and Dorri et al.^3^ found no significant difference in thyroid hormone levels before and after RT in breast cancer patients.

Although radiation‐induced thyroid disorders remain underestimated and study results are often contradictory, the current consensus is that RT causes hypothyroidism, and *V*_30_ and *D*_mean_ values have the most predictive value for development of hypothyroidism in patients with breast cancer. Therefore, in our study, these two parameters were used as a reference for DC of the thyroid gland.

In the present study, all treatment plans provided adequate coverage of the planning target volume. Our results of IMRT_DC(−)_, HT_DC(−)_, IMRT_DC(+)_, and HT_DC(+)_ plans presented similar dosimetric results as the previous studies with respect to critical organs (e.g., contralateral breast, heart, and both lungs). The TD_mean_ > 21 Gy and the *V*_30_ was >50% for the FinF technique, which was not planned to include a special shield to reduce the dose to the thyroid gland. In the IMRT_DC(−)_ and HT_DC(−)_ plans, the TD_mean_ was >21 Gy, while the V_30_ was <50%. For the IMRT_DC(+)_ and HT_DC(+)_ plans, we were able to achieve the dose limits to the thyroid gland that we set for *V*_30_ and *D*_mean_.

In addition to dose‐volume parameters, other factors have been identified as predictors for thyroid dysfunction such as thyroid gland volume. Thus, accurate estimation of the size and localization of the thyroid is critical for evaluating dose‐volume parameters and management of thyroid disorders. Therefore, it is recommended that the thyroid gland is contoured by experienced radiation oncologists, and contrast‐enhanced CT may be beneficial.

One of the important challenges to address during breast RT is secondary cancer risk. Various reports have shown that increased low doses may increase the risk of secondary malignancy development.^36^, ^37^, ^38^ The move from three‐dimensional conformal RT to intensity‐modulated techniques involves more fields, and the dose‐volume histograms show that, as a consequence, a larger volume of normal tissue is exposed to lower doses. In addition, the number of monitor units is increase. Both factors will tend to increase the risk of development of secondary cancers. In this study, low dose‐volume (*V*_10_) was significantly larger in the IMRT_DC(−)_ and HT_DC(−)_ plans than in the FinF, IMRT_DC(+)_, and HT_DC(+)_ plans. According to some authors, *V*_10_ was not associated with hypothyroidism.^12^, ^35^ However, it should not be ignored that larger low dose‐volume may be a risk factor for the development of secondary thyroid cancer in breast cancer patients with long life expectancies.

## 5 CONCLUSION

The use of intensity‐modulated techniques with thyroid DC_(+)_ significantly reduce the dose to the thyroid gland when compared with DC_(−)_ for the breast patients with SCF irradiation; therefore, it is recommended that recognition of the thyroid as an OAR and the use of DCs during IMRT and HT planning to minimize radiation dose and thyroid volume. Future clinical studies are needed to confirm this dosimetric results.

## CONFLICT OF INTEREST

The authors declare that there is no conflict of interest regarding the publication of this article.
